# Longitudinal Metabolomics Reveals Metabolic Dysregulation Dynamics in Patients with Severe COVID-19

**DOI:** 10.3390/metabo14120656

**Published:** 2024-11-25

**Authors:** Ryo Uchimido, Kenjiro Kami, Hiroyuki Yamamoto, Ryo Yokoe, Issei Tsuchiya, Yoko Nukui, Yuki Goto, Mariko Hanafusa, Takeo Fujiwara, Kenji Wakabayashi

**Affiliations:** 1 Department of Intensive Care Medicine, Institute of Science Tokyo, 1-5-45 Yushima, Bunkyo City 113-8510, Japan; uchrccm@tmd.ac.jp (R.U.);; 2 Human Metabolome Technologies, Inc., 246-2 Mizukami Kakuganji, Tsuruoka City 997-0052, Japan; 3 Department of Infection Control and Laboratory Medicine, Kyoto Prefectural University of Medicine, Kamigyo-ku Kajii-cho, Kawaramachi-Hirokoji, Kyoto 602-8566, Japan; 4 Department of Tokyo Metropolitan Health Policy Advisement, Institute of Science Tokyo, 1-5-45 Yushima, Bunkyo City 113-8519, Japan; 5 Division of Cohort Research, National Cancer Center Institute for Cancer Control, Tokyo 104-0045, Japan; 6 Department of Public Health, Institute of Science Tokyo, 1-5-45 Yushima, Bunkyo City 113-8519, Japan

**Keywords:** longitudinal metabolic dysregulation, severe COVID-19

## Abstract

**Background/Objective:** A dysregulated metabolism has been studied as a key aspect of the COVID-19 pathophysiology, but its longitudinal progression in severe cases remains unclear. In this study, we aimed to investigate metabolic dysregulation over time in patients with severe COVID-19 requiring mechanical ventilation (MV). **Methods:** In this single-center, prospective, observational study, we obtained 236 serum samples from 118 adult patients on MV in an ICU. The metabolite measurements were performed using capillary electrophoresis Fourier transform mass spectrometry, and we categorized the sampling time points into three time zones to align them with the disease progression: time zone 1 (T1) (the hyperacute phase, days 1–3 post-MV initiation), T2 (the acute phase, days 4–14), and T3 (the chronic phase, days 15–30). Using volcano plots and enrichment pathway analyses, we identified the differential metabolites (DMs) and enriched pathways (EPs) between the survivors and non-survivors for each time zone. The DMs and EPs were further grouped into early-stage, late-stage, and consistent groups based on the time zones in which they were detected. **Results:** With the 566 annotated metabolites, we identified 38 DMs and 17 EPs as the early-stage group, which indicated enhanced energy production in glucose, amino acid, and fatty acid metabolisms in non-survivors. As the late-stage group, 84 DMs and 10 EPs showed upregulated sphingolipid, taurine, and tryptophan–kynurenine metabolisms with downregulated steroid hormone synthesis in non-survivors. Three DMs and 23 EPs in the consistent group showed more pronounced dysregulation in the dopamine and arachidonic acid metabolisms across all three time zones in non-survivors. **Conclusions:** This study elucidated the temporal differences in metabolic dysregulation between survivors and non-survivors of severe COVID-19, offering insights into its longitudinal progression and disease mechanisms.

## 1. Introduction

Dysregulated metabolism has been recognized as a crucial feature in critical illnesses [1], such as severe coronavirus infectious disease (COVID-19), which is characterized by multiple organ failures due to dysregulated systemic responses of the host to SARS-CoV-2 infection [2,3,4]. Despite advances in supportive care and management for severe COVID-19, there has been no substantial improvement in the mortality in intensive care units (ICUs), which is persistently high at about 20–30% [5,6].

COVID-19-associated metabolic dysregulation includes abnormal energy production through glucose, lipid, and amino acid metabolisms, which can drive excessive inflammatory and oxidative responses [7,8,9]. Specifically, excessive glycolysis, fatty acid (FA) oxidation, and amino acid catabolisms have been associated with the overproduction of inflammatory cytokines and reactive oxygen species (ROS), contributing to tissue and organ damage [10,11,12,13]. Additionally, increased kynurenine suppresses the pro-inflammatory response via NF-κB inhibition, while decreased dopamine may undermine the anti-viral activity by downregulating type-I interferons (IFNs). Such metabolic disruptions could impair the immune defenses and exacerbate tissue damage [14,15,16]. Moreover, these metabolic dysregulations can promote thromboembolic events by facilitating excessive platelet aggregation, disseminated intravascular coagulopathy, and endothelial dysfunction [17,18,19]. These pathophysiological interactions further suggest that metabolic dysregulations can be a key factor in post-COVID-19 syndrome, which presents with fatigue, dyspnea, immobility, cognitive impairment, and depression [20].

Most metabolomics studies that have characterized metabolic dysregulations in patients with severe COVID-19 have compared them with mild and moderate cases at a single time point. In these studies, it was discovered that most metabolic abnormalities in patients with severe COVID-19 represent amplifications of those with a mild and moderate disease; however, some of them involve distinct pathophysiological entities rather than mere amplifications. In previous studies, the blood steroid hormone levels increased with the severity from healthy to mild and moderate cases but decreased in severe cases [21,22], which may be attributed to the ischemic necrosis of the adrenal glands, a pathologic condition unique to patients with severe COVID-19 [23]; however, this area remains largely unexplored.

Serial metabolomic analyses have recently been used to describe the metabolic dysregulation trajectories in COVID-19 patient cohorts of varying severity [24,25,26]. However, this approach has rarely been applied to cohorts of patients with severe COVID-19. Hence, which metabolic dysregulations improve or persist following the initiation of intensive care and which new metabolic dysregulations emerge after the initial treatment are poorly understood. Given that the ICU mortality is a critical outcome in clinical settings, characterizing the temporal differences in the metabolic states between survivors and non-survivors could help elucidate the trajectory of metabolic dysregulation that is both critical and prognostic in patients with severe COVID-19. This characterization may assist in identifying the optimal timings for interventions targeting metabolic dysregulation in patients with severe COVID-19. Therefore, in the present study, we investigated the longitudinal difference in metabolic dysregulation between survivors and non-survivors among patients with severe COVID-19.

## 2. Materials and Methods

### 2.1. Study Design and Participants

This study was a single-center, prospective, and observational study conducted between 4/2020 and 10/2021 in an intensive care unit (ICU) at a tertiary university hospital. Patients aged >18 years requiring mechanical ventilation (MV) due to COVID-19, confirmed via a polymerase chain reaction test, were eligible for this study. To account for the substantial effect of the sedative and muscle relaxant drugs used in the endotracheal intubation procedures and MV on the human metabolome [27], we excluded patients who did not require mechanical ventilation. All the statistical analyses were performed using R software (version 4.3.1, R Foundation for Statistical Computing, Vienna, Austria), and the *p*-values were adjusted using the Benjamini–Hochberg (BH) method when needed.

### 2.2. Blood Sample Collection

The remaining serum samples utilized for routine laboratory testing were collected and subsequently stored at −80 °C. We obtained these samples from patients during their stays both in the ICU and in wards, when possible. As a result, we collected samples from 118 patients (93 survivors and 25 non-survivors). We classified the sampling timing into three time zones to align them with the disease progression: time zone 1 (T1), which ranged from 1 to 3 days after the MV initiation, representing the hyperacute phase wherein immediate pathophysiological changes are the most pronounced; time zone 2 (T2), covering days 4 to 14, defined as the acute phase, capturing the ongoing patient status with the treatment effect; and time zone 3 (T3), spanning from 15 to 30 days, marking the chronic phase wherein longer-term maladaptive responses are observed [28]. We defined day 1 as the day when endotracheal intubation and MV were conducted because they substantially affect the human metabolome [27]. We collected a total of 236 samples: 73 in T1 (56 from survivors and 17 from non-survivors), 107 in T2 (82 from survivors and 25 from non-survivors), and 56 in T3 (35 from survivors and 21 from non-survivors).

### 2.3. Metabolome Measurements

We conducted the metabolite extraction and metabolome analysis at Human Metabolome Technologies, Inc. (HMT, Tsuruoka, Yamagata, Japan). Before the metabolite extraction, the inactivation of SARS-CoV-2 was undertaken by adding methanol to the serum. Two types of targeted metabolomics profiling were conducted: the Omega Scan and LC-Omega Scan.

In the Omega Scan, 50 µL of serum was added to 200 µL of methanol containing internal standards (H3304-1002, HMT) at 0 °C to suppress the viral and enzymatic activities for the metabolite extraction, which was conducted at Kotobiken Medical Laboratories, Inc. (Iwaki, Fukushima, Japan). The extracted solution was thoroughly mixed with 150 µL of Milli-Q water, after which 300 µL of the mixture was centrifugally filtered through a Millipore 5 kDa cutoff filter (ULTRAFREE MC PLHCC, HMT) at 9100× *g* and 4 °C for 120 min to remove the macromolecules. The filtrate was then evaporated to dryness under a vacuum and reconstituted in 50 µL of Milli-Q water for the metabolome analysis at HMT. We used capillary electrophoresis Fourier transform mass spectrometry (CE-FTMS) based on the previously described methods [29]. Briefly, the CE-FTMS analysis was conducted using an Agilent 7100 CE capillary electrophoresis system equipped with a Q Exactive Plus (Thermo Fisher Scientific Inc., Waltham, MA, USA), an Agilent 1260 isocratic HPLC pump, an Agilent G1603A CE-MS adapter kit, and an Agilent G1607A CE-ESI-MS sprayer kit (Agilent Technologies, Inc., Santa Clara, CA, USA). The systems were controlled via the Agilent MassHunter workstation software LC/MS data acquisition for 6200 series TOF/6500 series Q-TOF version B.08.00 (Agilent Technologies) and Xcalibur (Thermo Fisher Scientific) and were connected by a fused silica capillary (total length: 50 μm i.d. × 80 cm) with a commercial electrophoresis buffer (H3301-1001 and I3302-1023 for the cation and anion analyses, respectively, at HMT) as the electrolyte. The spectrometer was scanned from m/z 60 to 900 in positive mode, and from m/z 70 to 1050 in negative mode [29]. The peaks were extracted using MasterHands automatic integration software (Keio University, Tsuruoka, Yamagata, Japan) to obtain the peak information, including the m/z, peak area, and migration time (MT) [30]. Signal peaks corresponding to isotopomers, adduct ions, and other product ions of the known metabolites were excluded, and the remaining peaks were annotated according to HMT’s metabolite database based on their m/z values and MTs. The areas of the annotated peaks were then normalized to internal standards and the sample volume in order to obtain the relative levels of each metabolite.

In the LC-Omega Scan, 100 µL of serum was added to 300 µL of 1% formic acid/acetonitrile containing internal standards (H3304-1002, HMT) at 0 °C to suppress the viral and enzymatic activities for the metabolite extraction, which was conducted at Kotobiken Medical Laboratories, Inc. The mixture was centrifuged at 2300× *g* and 4 °C for 5 min and then filtered using a hybrid SPE phospholipid cartridge (hybrid SPE–phospholipids, 30 mg/mL, SUPELCO, PA, USA) to remove the phospholipids. Subsequently, the filtrate was evaporated to dryness under nitrogen and reconstituted in 100 µL of 50% isopropanol (v/v) for the metabolome analysis at HMT. We used liquid chromatography Fourier transform mass spectrometry (LC-FTMS) based on the methods described previously [31,32]. Briefly, the LC-FTMS analysis was performed using the Vanquish Flex UHPLC System (Thermo Fisher Scientific). The systems were controlled using Xcalibur (Thermo Fisher Scientific) and were connected by an ODS column (2 mm i.d. × 50 mm, 2 μm). The spectrometer was scanned from m/z 100 to 1500, and the peaks were extracted using the same software as that used for the Omega Scan [30].

### 2.4. Clinical Parameters and Laboratory Test Measurements

We collected patient demographics and clinical parameters, including age, sex, and BMI; comorbidities such as hypertension, diabetes mellitus, dyslipidemia, asthma, COPD, and cancer; severity scores upon ICU admission such as APACHE II and SOFA; medications and treatments including steroids, favipiravir, remdesivir, tocilizumab, steroid pulse therapy, renal replacement therapy, and extracorporeal membrane oxygenation; complications such as ventilator-associated pneumonia, bloodstream infections, and pneumothorax; and clinically relevant intervals such as time from intubation to ICU discharge and the length of ICU stay. We collected the following laboratory test results: the white blood cell count; lymphocyte count; C-reactive protein (CRP); neutrophil–lymphocyte ratio (NLR); hemoglobin; platelet count; mean platelet volume (MPV); albumin; aspartate aminotransferase (AST); alanine aminotransferase (ALT); total bilirubin; γ-glutamyl transpeptidase (GTP); lactate dehydrogenase (LDH); creatine kinase (CK); creatinine; sodium; potassium; chloride; calcium; magnesium; phosphate; D-dimers; fibrinogen; antithrombin (AT); prothrombin time-international normalized ratio (PT-INR); activated partial thromboplastin time (APTT); P/F ratio; partial pressure of carbon dioxide (PaCO_2_); alveolar–arterial oxygen gradient (pO_2_(A-a) gradient); pH; bicarbonate (HCO_3_^−^); and base excess (BE). Using summary statistics appropriately, we described the baseline patient characteristics and compared the survivors and non-survivors in the ICU, and we analyzed the laboratory test results at each time zone in the same comparison.

### 2.5. PCA and PLS-ROG Analysis

For the metabolome data processing before the analysis, we removed metabolites with missing values over 50%, imputed the missing values with one-fifth of the minimum detected metabolite value, and scaled the metabolite levels. Initially, we conducted both the principal component (PCA) and partial least squares with the rank order of groups (PLS-ROG) analyses [33]. While PCA is an unsupervised learning method that does not account for any groups, PLS-ROG analysis is a novel method that differentiates groups and reflects their rank order, enabling the investigation of the association between the metabolic status and time-dependent subclasses, such as the survivors and non-survivors at T1, T2, and T3. In our PLS-ROG analysis, we set the time-dependent subclasses as the response variables and the metabolite levels as the exploratory variables, calculating the PLS-ROG scores for both. To illustrate the longitudinal metabolic status, we plotted the average trajectories of the survivors and non-survivors using the PLS-ROG 1 and 2 scores of the metabolite levels, and we then conducted hypothesis testing on the factor loading in the PLS-ROG 1 and 2 scores to select metabolites significantly correlated with the time-dependent subclasses. This selection was based on the Pearson’s correlation coefficients between the PLS-ROG scores of the time-dependent subclasses and metabolite levels.

### 2.6. Selection of Differential Metabolites and Enriched Pathways

We used a volcano plot analysis to select differential metabolites (DMs) between the survivors and non-survivors at each time zone. Each metabolite was tested using a *t*-test and chosen based on two criteria: (1) a BH-adjusted *p*-value < 0.05 and (2) an absolute log2-fold change > 1. The DMs were categorized into early-stage, late-stage, and consistent groups based on the time zones in which they were detected. The early-stage DMs included those found only in T1, in both T1 and T2, and only in T2, the late-stage DMs were those found in both T2 and T3 or only in T3, and the consistent DMs were those identified across all three time zones: T1, T2, and T3. We did not name or further analyze the DMs found in T1 and T3 due to the difficulty in the longitudinal interpretation. For the enrichment pathway analysis, we used quantitative enrichment analysis in MetaboAnalyst 6.0., defining the pathways with false discovery rates (FDRs) of <0.05 between the survivors and non-survivors as enriched pathways (EPs). Similar to the DMs, we classified the EPs into early-stage, late-stage, and consistent groups, and we described the temporal changes in the pathway enrichment levels and FDRs across the time zones using dot plots.

### 2.7. Linear Mixed-Effects Model

To examine the association between the repeated measures of the metabolite levels and ICU outcomes, we constructed a linear mixed-effects model, adjusting it for the age, sex, and time zone. Our model included fixed effects for the age, sex, ICU outcome, and time zones as categorical variables for repeated measurements, an interaction term for the ICU outcomes and time zones, and the random effects of individual subjects.

### 2.8. Prognostic Ability

To assess the prognostic ability of the consistent DMs for the ICU outcomes over time, we calculated the area under the receiver operating characteristic curve (AUC) for each consistent DM. Additionally, we developed a logistic regression model including age and sex for the consistent DMs at each time zone to further evaluate their predictive value.

### 2.9. Correlation Analysis

**Figure 1:**
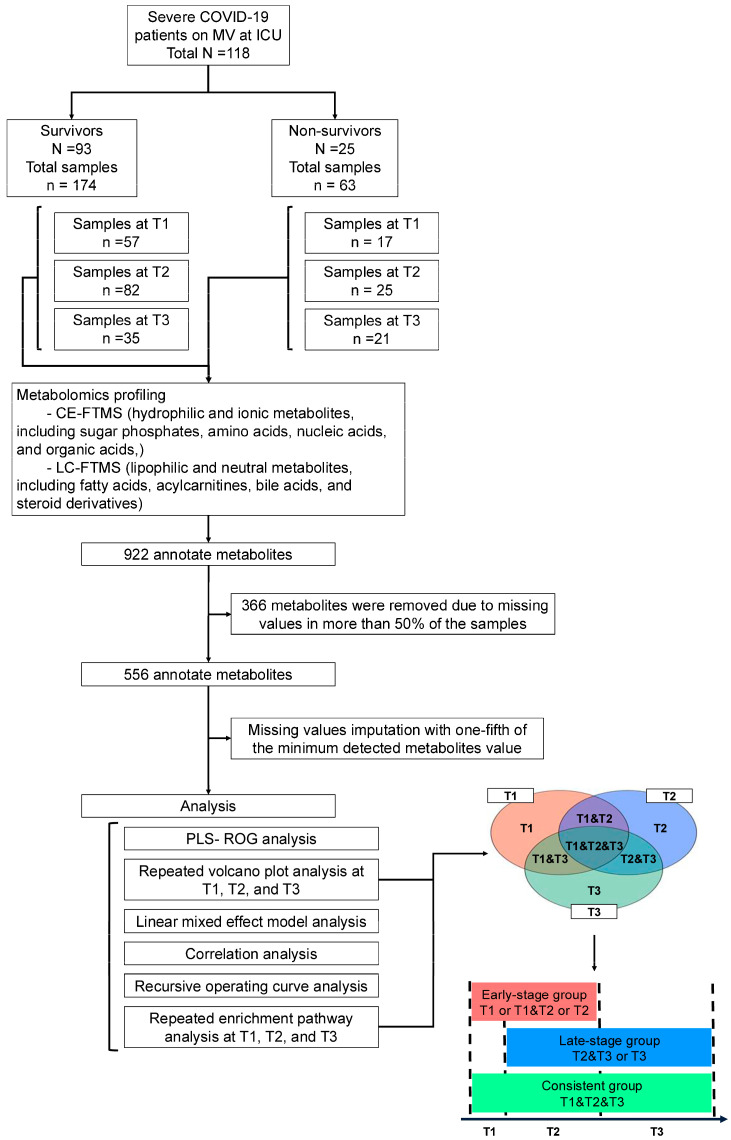
Workflow of the metabolomics analysis. N represents the number of patients, and n represents the number of samples. PLS-ROG: partial least square with ranking of groups; T1: time zone 1; T2: time zone 2; T3: time zone 3.

We explored the correlation between the laboratory test results and DMs to gain clinical insights. Spearman’s correlation coefficients were calculated between the laboratory values and metabolite levels, and heatmaps of the correlation matrices were created with hierarchical clustering. The workflow of the metabolomics analysis is shown in Figure 1.

## 3. Results

### 3.1. Patient Characteristics

In this cohort of 118 patients, comprising 93 survivors and 25 non-survivors, the median age was 58 years for the survivors compared to 71 years for the non-survivors, and 18% of the survivors and 20% of the non-survivors were females. The median BMI was 24.7 kg/m^2^ in the survivors and 24.0 kg/m^2^ in the non-survivors. Comorbidities, including hypertension, diabetes mellitus, ischemic heart disease, congestive heart failure, hemodialysis, and interstitial pneumonia, were more prevalent among the non-survivors (Table 1). Table 1 shows that the non-survivors also had higher median APACHE II and SOFA scores. Regarding the treatment modalities, the non-survivors had higher nitric oxide therapy, prone positioning, extracorporeal membrane oxygenation, and renal replacement therapy utilization rates. Complications such as arrhythmia, mediastinal emphysema, ventilator-associated pneumonia, and bloodstream infections were more frequent in the non-survivors. Additionally, the median interval from intubation to ICU discharge was longer for the non-survivors, and a greater proportion of non-survivors had ICU stays exceeding 14 days.

### 3.2. PLS-ROG Analysis Showed Longitudinal Differences in Metabolic Dysregulation Between Survivors and Non-Survivors

**Figure 2:**
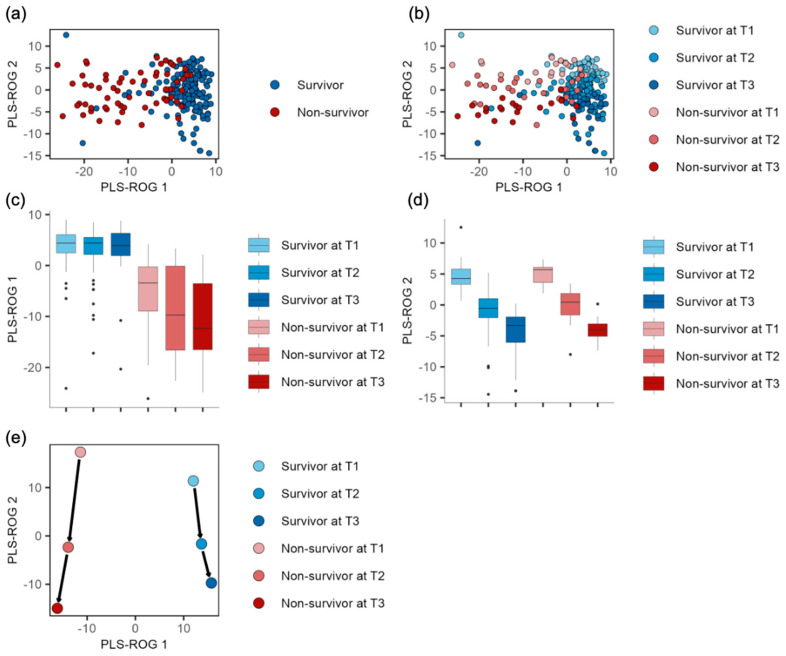
Partial least squares with rank order of groups (PLS−ROG) analysis revealed the association between the metabolic profiles and time−dependent subclasses. (**a**) PLS−ROG analysis illustrating the distribution of the first and second PLS−ROG scores for the metabolite levels as the explanatory variables in the model, with the coloring based on the ICU outcomes. (**b**) PLS−ROG analysis demonstrating the distribution of the first and second PLS−ROG scores, with the colors representing the time−dependent subclasses. (**c**,**d**) Box plots displaying the first and second PLS−ROG scores across the different time−dependent subclasses, respectively. (**e**) Dot plot with arrows showing the trajectories of the mean metabolic profiles with each time−dependent subclass.

A total of 566 annotated metabolites were included in our analysis. To comprehensively explore and visualize the longitudinal differences in the metabolic dysregulation between the survivors and non-survivors, we first performed a principal component analysis (PCA), which distinctly clustered the survivors and non-survivors (Figure S1a). However, because the PCA did not differentiate the time-dependent subclasses (Figure S1b), we also employed partial least squares with the rank order of groups (PLS-ROG) analyses, the modeling of which allowed us to create a scatter plot with the first and second PLS-ROG scores for the metabolite levels as the explanatory variables in the model. In this plot, the PLS-ROG analyses successfully clustered the survivors and non-survivors (Figure 2a) and differentiated the time-dependent subclasses (Figure 2b). We observed associations between the PLS-ROG1 scores and ICU outcomes (Figure 2c) and between the PLS-ROG2 scores and time-dependent subclasses (Figure 2d). In addition, the PLS-ROG analysis depicted the distinct trajectories of the average metabolic profiles between the survivors and non-survivors (Figure 2e). A scatter plot of the first and second PLS-ROG scores for the time-dependent subclasses as the response variables in the model also distinctly clustered the ICU outcomes and time-dependent subclasses (Figure S2a–c). Using PLS-ROG loadings based on Pearson’s correlation coefficients between the PLS-ROG1 scores for the time-dependent subclasses and metabolite levels, we identified 54 metabolites with statistically significant correlation coefficients. Among these, the top five ranked by their absolute correlation coefficients were kynurenine, 1-methyladenosine, homovanillic acid (HVA), quinolinic acid, and *N*1-Methyl-4-pyridone-5-carboxamide (Figure S3a). Similarly, using Pearson’s correlation coefficients between the PLS-ROG2 scores for the time-dependent subclasses and metabolite levels, we identified two statistically significant metabolites: hydroxyproline and hydroxybutyric acid (Figure S3b).

### 3.3. Repeated Volcano Plot Analysis Identified Differential Metabolites Between Survivors and Non-Survivors at Each Time Zone

**Figure 3:**
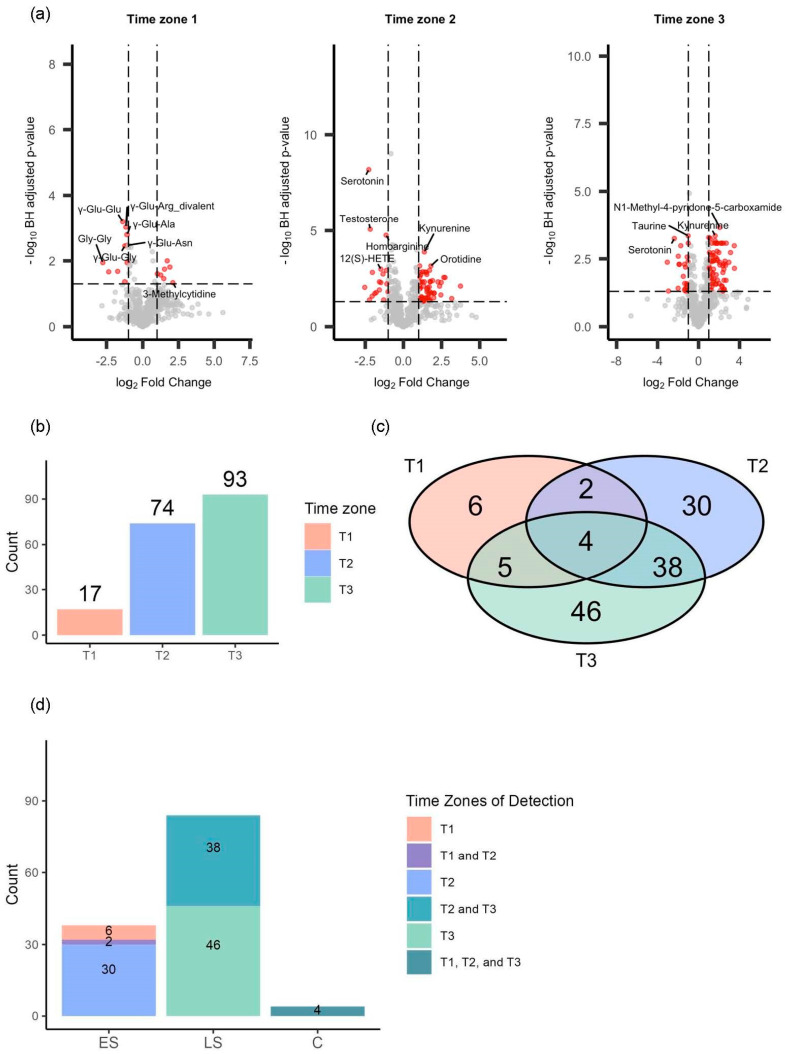
Change in differences in metabolic dysregulation between survivors and non−survivors over time. (**a**) Three volcano plots displaying significantly differential metabolites (DMs) between survivors and non−survivors with the red color at each time zone, wherein each dot represents a metabolite. (**b**) Bar charts showing the number of DMs at each time zone: 17 in time zone 1 (T1), 74 in time zone 2 (T2), and 93 in time zone 3 (T3). (**c**) Venn diagram illustrating the distribution of DMs identified across different time zone combinations: only in T1; in both T1 and T2; in both T1 and T3; only in T2; in both T2 and T3; only in T3; and in all three time zones (T1, T2, and T3). (**d**) Bar plots presenting the number of DMs across the different time zone combinations, categorized into early−stage, late−stage, and consistent DMs. The 38 early−stage DMs were found either only in T1 (n = 6), in both T1 and T2 (n = 2), or only in T2 (n = 30); 84 late−stage DMs were identified either only in T2 (n = 38) or in both T2 and T3 (n = 46); and four consistent DMs were detected across all three time zones: T1, T2, and T3 (n = 4).

**Figure 4:**
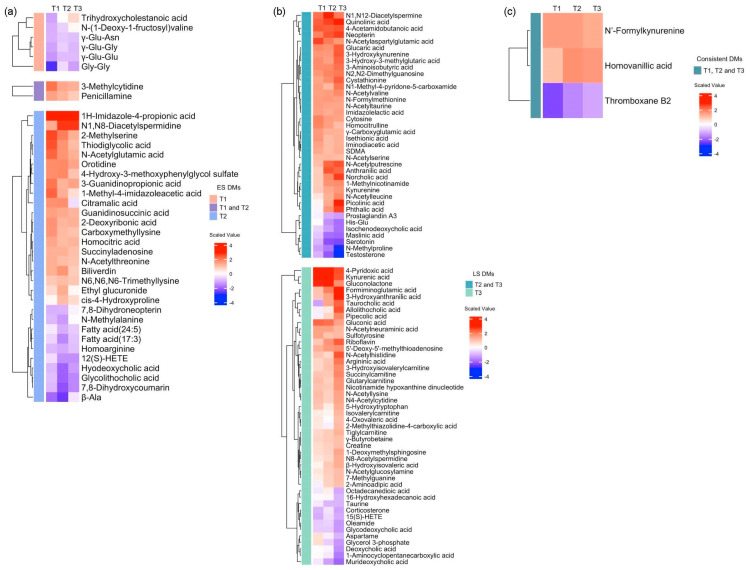
Heatmaps depicting the log2 fold changes (log2FCs) in the metabolite values when comparing non−survivors to survivors for (**a**) early−stage DMs (n = 38), (**b**) late−stage DMs (n = 84), and (**c**) consistent DMs (n = 4).

To investigate the longitudinal difference in the metabolic dysregulation between the survivors and non-survivors, we conducted repeated volcano plot analysis, identifying DMs at each time zone: 18 in T1, 75 in T2, and 94 in T3 (Figure 3a,b). All the volcano plot analyses are shown in Table S1, and heatmaps with hierarchical clustering depict the list of DMs at each time zone (Figure 4a–c). Based on our categorization, we identified 38 early-stage, 84 late-stage, and 4 consistent DMs (Figure 3c,d). The heatmaps in Figure 4a–c illustrate the log2 fold changes (log2FCs) of all the DMs classified in these groups, and the time series box plots display the longitudinal changes in the DM levels, as well as the temporal variation in the significant differences between the survivors and non-survivors across all three time zones.

**Figure 5:**
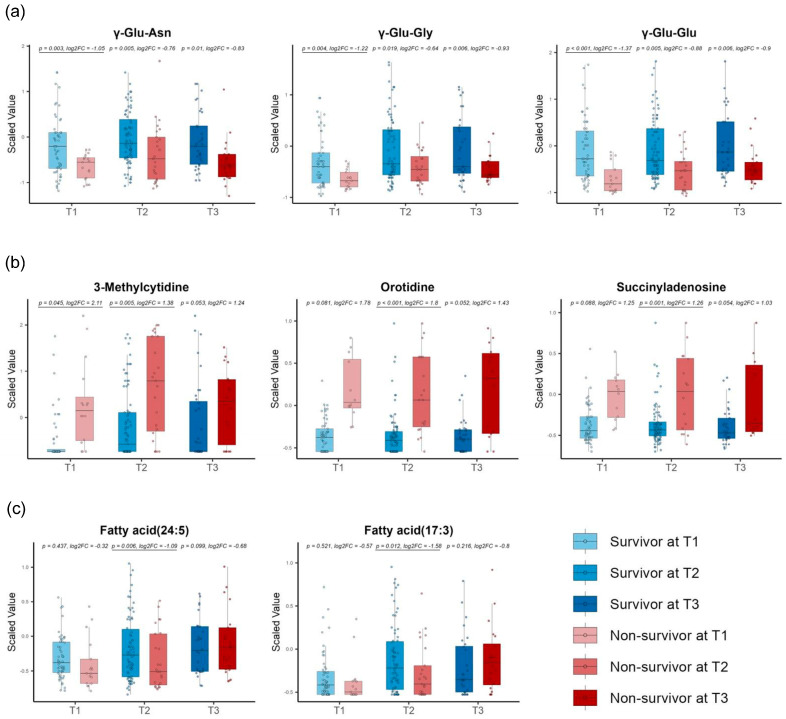
Time−series box plots showing the longitudinal changes in the scaled values of the early−stage differential metabolites and the temporal variation in the significant differences between the survivors and non-survivors across all three time zones. (**a**) The γ−glutamyl dipeptides γ−Glu−Asn, γ−Glu−Gly, and γ−Glu−Glu. (**b**) Metabolites related to nucleic acid metabolism, such as 3-methylcytidine, orotidine, and succinyl adenosine. (**c**) FA (24:5) and FA (17:3) involved in lipid metabolism. All *p*−values were adjusted using the Benjamini−Hochberg method. Underlined numbers met both criteria for differential metabolites: (1) a BH−adjusted *p*−value of <0.05 and (2) an absolute log2 fold change of >1.

Among the early-stage DMs, we found dipeptides involved in the γ-glutamyl cycles for the glutathione biosynthesis and degradation, such as γ-Glu-Asn, γ-Glu-Gly, and γ-Glu-Glu (Figure 5a). The early-stage DMs also included metabolites related to nucleic acid metabolism, such as 3-methylcytidine, orotidine, and succinyl adenosine (Figure 5b). In addition, we identified metabolites involved in lipid metabolism, such as FA (24:5) and FA (17:3) in T2 (Figure 5c).

**Figure 6:**
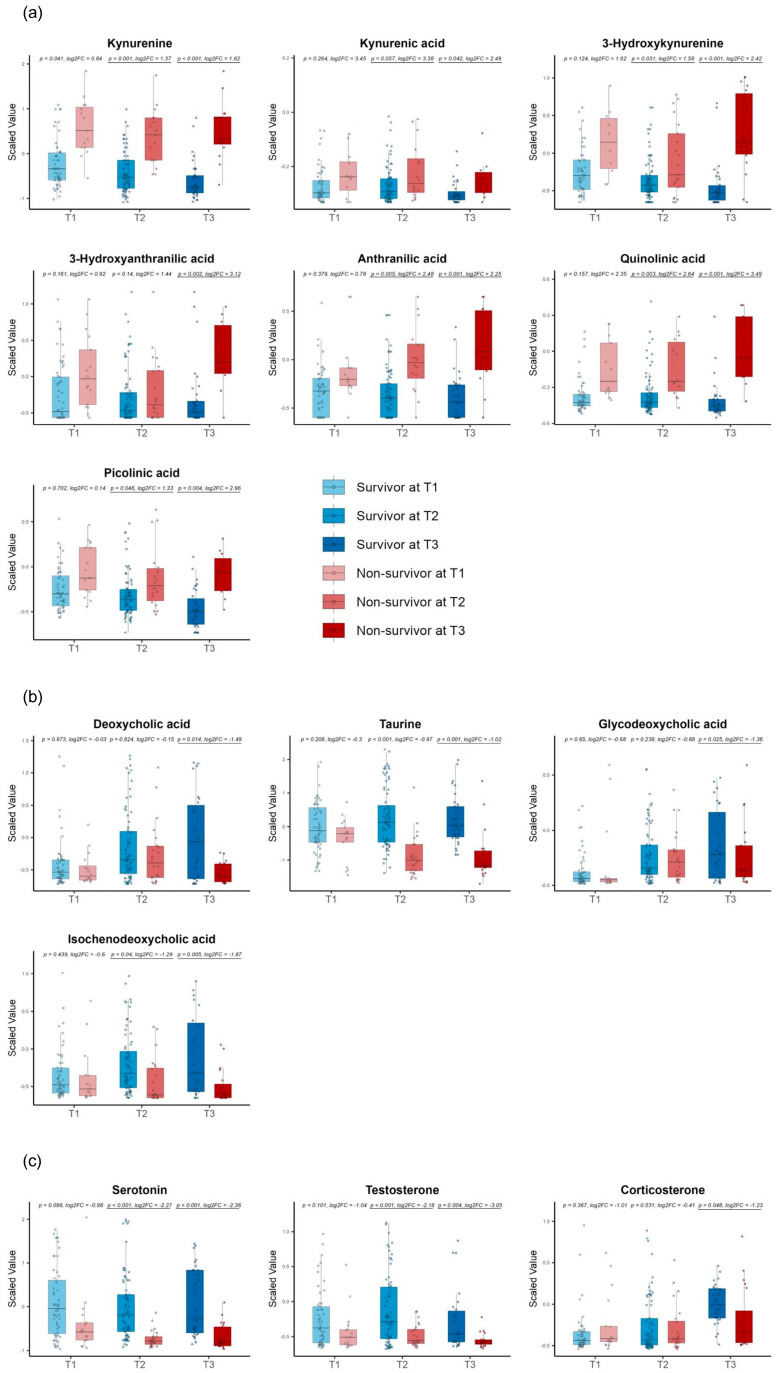
Time−series box plots showing the longitudinal changes in the scaled values of the late−stage differential metabolites and the temporal variation in the significant differences between the survivors and non−survivors across all three time zones. (**a**) Metabolites from the kynurenine pathway, including kynurenine, kynurenic acid, anthranilic acid, 3−hydroxy kynurenine, 3−hydroxy anthranilic acid, quinolinic acid, and picolinic acid. (**b**) Bile acid metabolism metabolites, such as deoxycholic acid, taurine, glycodeoxycholic acid, and isochenodeoxycholic acid. (**c**) Hormones, including serotonin, testosterone, and corticosterone. All *p*−values were adjusted using the Benjamini−Hochberg method. Underlined numbers met both criteria for differential metabolites: (1) a BH−adjusted *p*−value of <0.05 and (2) an absolute log2 fold change of >1.

For the late-stage DMs, we discovered metabolites from the kynurenine pathway, such as kynurenine, kynurenic acid, anthranilic acid, 3-hydroxy kynurenine, 3-hydroxy anthranilic acid, quinolinic acid, and picolinic acid (Figure 6a). We also found several metabolites involved in bile acid metabolism, such as deoxycholic acid, taurine, glycodeoxycholic acid, and isochenodeoxycholic acid (Figure 6b), and we, furthermore, detected serotonin as a neurotransmitter and testosterone and corticosterone as hormones (Figure 6c).

**Figure 7:**
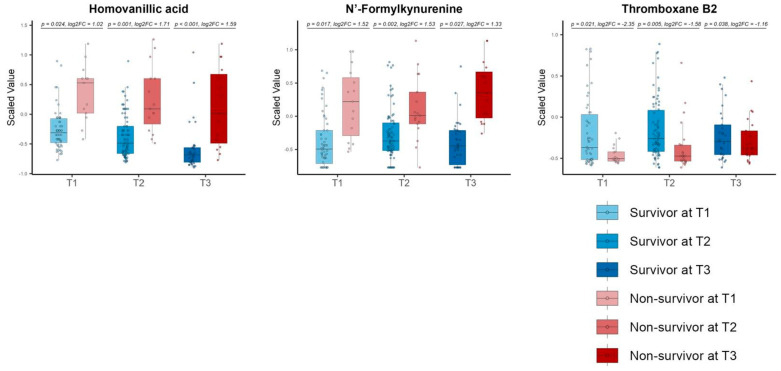
Time−series box plots showing the longitudinal changes in the scaled values of the consistent differential metabolites and the temporal variation in the significant differences between the survivors and non−survivors across all three time zones. The plots show homovanillic acid, *N*′−formyl kynurenine, and thromboxane B2. All *p*−values were adjusted using the Benjamini−Hochberg method. Underlined numbers met both criteria for differential metabolites: (1) a BH−adjusted *p*−value of <0.05 and (2) an absolute log2 fold change of >1.

After excluding phenoxyacetic acid due to its drug origin, the consistent DMs comprised three metabolites: HVA, *N*’-formyl kynurenine, phenoxy acetic acid, and thromboxane B2 (TXB2) (Figure 7).

### 3.4. Linear Mixed Models Detected Different Metabolite Trends Between Survivors and Non-Survivors over Time

**Figure 8:**
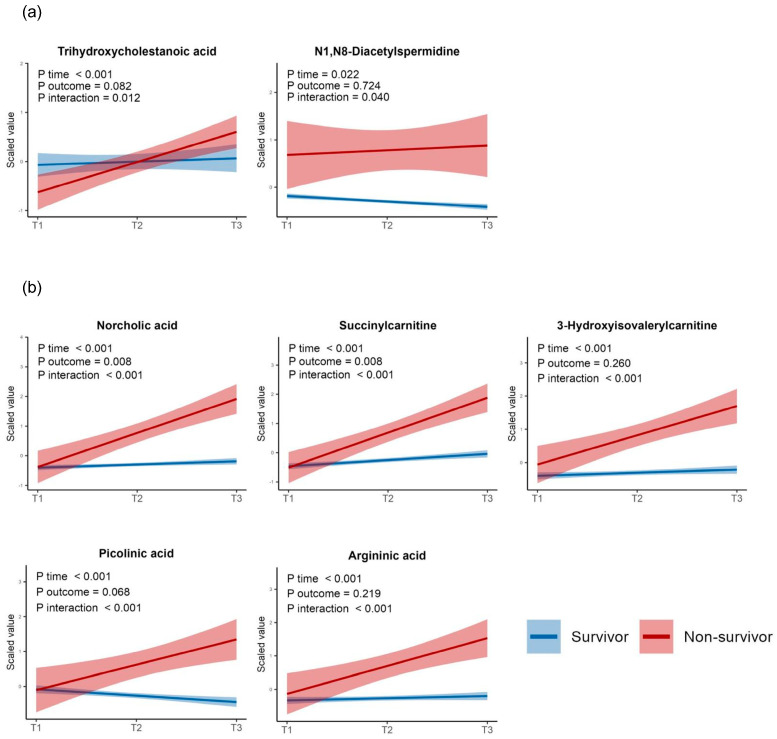
Linear mixed models displaying the *p*−values of the regression coefficients for the time zones, ICU outcomes (survival or not), and interaction terms between the time zones and ICU outcomes. (**a**) Two early−stage differential metabolites with significant *p*−values for the interaction term. (**b**) The top five metabolites with significant *p*-values for the interaction term among the late−stage differential metabolites. All *p*−values were adjusted using the Benjamini–Hochberg method.

To assess the differences in the trajectories of the DM levels between the survivors and non-survivors, we performed a linear mixed-model analysis and identified two early-stage DMs with significant trend differences: trihydroxy cholestanoic acid and *N*1, *N*8-diacetyl spermidine. Additionally, 38 late-stage DMs also showed significant trend differences. Of these, the top five metabolites ranked by their adjusted *p*-values were norcholic acid, succinyl carnitine, 3-hydroxyisovalerylcarnitine, picolinic acid, and argininic acid. In contrast, no metabolites showed significant trend differences among the consistent DMs (Figure 8a,b for the early- and late-stage DM categories, respectively). Moreover, we found 41 metabolites that were significantly associated with ICU mortality after adjusting for age, sex, the time zones, and the interactions between the time zones and metabolite levels. The results of the linear mixed-model analysis are presented in Table S2.

### 3.5. Correlation Analysis Discovered Correlations of Differential Metabolites with Clinical Laboratory Test Results

**Figure 9:**
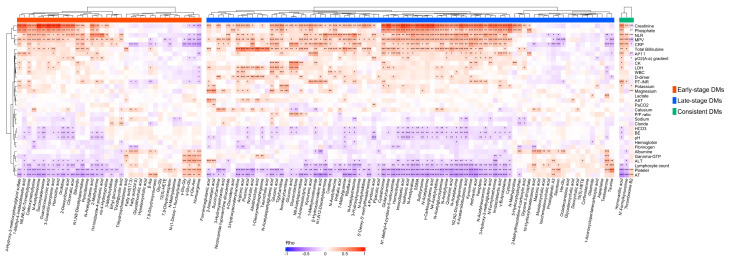
Correlation heatmap depicting the Spearman’s correlations between the differential metabolites and clinical laboratory data. All *p*−values were adjusted using the Benjamini−Hochberg method. Significance levels are indicated as follows: *** *p* < 0.001, ** *p*  < 0.01, and * *p*  < 0.05.

We investigated the correlations between the DMs and clinical laboratory data to obtain the clinical implications of DMs. In Figure 9, the heatmap with hierarchical clustering shows that most of the DMs were significantly correlated with the CRP, lymphocyte counts, neutrophil–lymphocyte ratio (NLR), platelet count, mean platelet volume (MPV), hemoglobin, and creatinine blood levels. Among the early-stage DMs, γ-glutamyl amino acids, including γ-Glu-Asn, γ-Glu-Glu, and γ-Glu-Gly, were significantly correlated with liver injury markers such as γ-GTP, ALT, and albumin. Most of the late-stage DMs were strongly correlated with the total bilirubin blood levels, and taurine demonstrated a significant correlation with the platelet count (*ρ* = 0.62). After excluding phenoxy acetic acid due to its drug origin, the consistent DMs showed weak but significant correlations with the laboratory data: HVA was correlated with creatinine (*ρ* = 0.58), AT (*ρ* = −0.53), PT-INR (*ρ* = 0.4), platelets (*ρ* = −0.41), CRP (*ρ* = 0.36), and the NLR (*ρ* = 0.34); *N’*-formyl kynurenine was correlated with the lymphocyte count (*ρ* = −0.21) and CRP (*ρ* = 0.29); and TXB2 was correlated with the platelet count (*ρ* = 0.19), MPV (*ρ* = −0.26), and AT (*ρ* = 0.25). Details on the clinical laboratory data are presented in Supplementary Table S3.

### 3.6. The Differential Metabolites in the Consistent Group Showed Consistently High Prognostic Relevance

**Figure 10:**
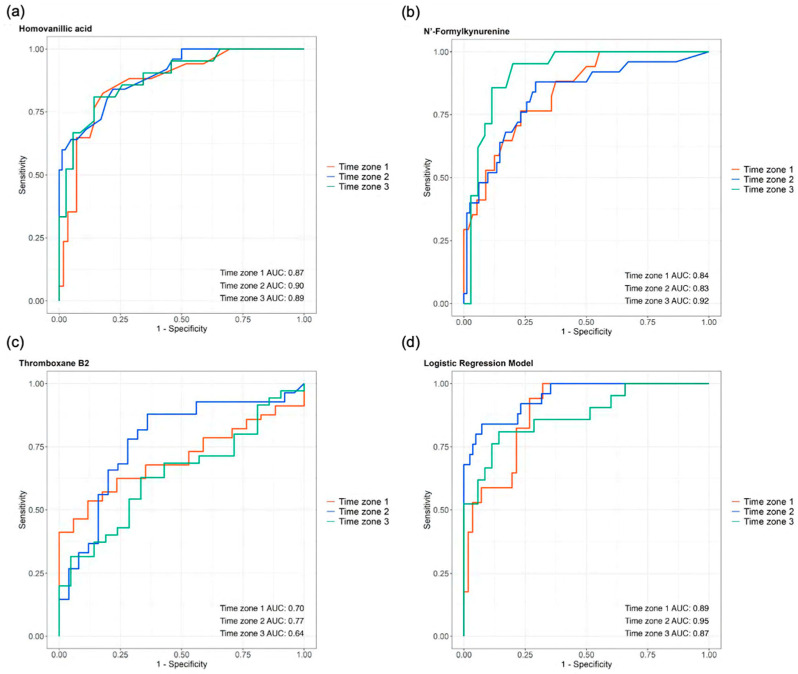
The prognostic abilities of homovanillic acid, N′-formylkynurenine, and thromboxane B2 demonstrated by the areas under the curves (AUCs) of their receiver operating characteristic (ROC) curves across all three time zones: (**a**) homovanillic acid AUCs: 0.87, 0.90, and 0.89; (**b**) N′-formylkynurenine AUCs: 0.84, 0.83, and 0.92; and (**c**) thromboxane B2 AUCs: 0.70, 0.77, and 0.64. (**d**) The logistic regression model, which incorporated these three metabolites along with the age and sex, showed AUCs of 0.89, 0.95, and 0.87 for time zones 1, 2, and 3, respectively.

We examined the predictive potential for the ICU outcomes of three consistent differential metabolites across all three time zones: HVA, *N′*-formyl kynurenine, and TXB2, which demonstrated high AUCs in all three zones: HVA: 0.87, 0.90, and 0.89; *N′*-formyl kynurenine: 0.84, 0.83, and 0.92; and TXB2: 0.70, 0.77, and 0.64 for T1, T2, and T3, respectively (Figure 10a, Figure 10b, and Figure 10c, respectively). We built a logistic regression model with these three metabolites using age and sex as the variables, which also showed high AUCs across all three time zones (0.89, 0.95, and 0.87 for time zones 1, 2, and 3, respectively) (Figure 10d).

### 3.7. Repeated Enriched Pathway Analysis Identified Enriched Pathways Between Survivors and Non-Survivors at Each Time Zone

**Figure 11:**
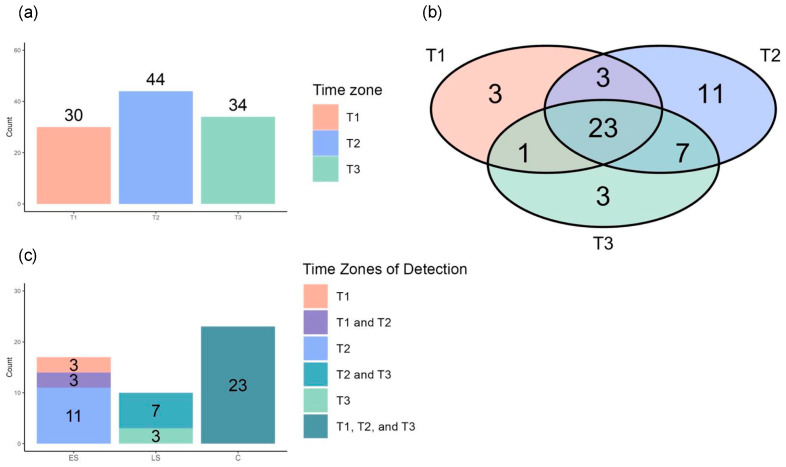
Enriched pathway analysis identified metabolic pathways enriched between survivors and non-survivors in each time zone. (**a**) Bar charts showing the number of enriched pathways (EPs) in each time zone: 30 in time zone 1 (T1), 44 in time zone 2 (T2), and 34 in time zone 3 (T3). (**b**) Venn diagram illustrating the distribution of EPs identified in the different time zone combinations: only in T1; in both T1 and T2; in both T1 and T3; only in T2; in both T2 and T3; only in T3; and in all three time zones (T1, T2, and T3). (**c**) Bar plots presenting the numbers of EPs across the different time zone combinations, categorized into early-stage, late-stage, and consistent EPs. The 17 early-stage EPs were found only in T1 (n = 3), in both T1 and T2 (n = 3), or only in T2 (n = 11); the 10 late-stage EPs were identified only in T2 (n = 7) or in both T2 and T3 (n = 3); and the 23 consistent EPs were detected across all three time zones: T1, T2, and T3 (n = 23).

We conducted an enriched pathway analysis for each time zone to further assess the longitudinal differences in the metabolic dysregulation between the survivors and non-survivors, detecting 30, 44, and 33 EPs in T1, T2, and T3, respectively (Figure 11a). Using the same method as that for the DM categorization, we identified 17 early-stage, 10 late-stage, and 23 consistent EPs (Figure 11b,c).

In the early-stage EPs, we found several glucose metabolism pathways involved in ATP production, including galactose metabolism, the citrate cycle, glycolysis/gluconeogenesis, and pyruvate metabolism. We also detected lipid metabolism pathways, including FA degradation, ether lipid, glycerolipid, arachidonic acid, and linoleic acid metabolism, and FA elongation. Additionally, we observed several amino acid metabolism pathways, such as those for phenylalanine, tyrosine, and tryptophan biosynthesis and those for the degradation and biosynthesis of valine, leucine, and isoleucine.

In the late-stage EPs, we observed several pathways related to immunomodulation and inflammation, such as sphingolipid metabolism, taurine and hypotaurine metabolism, and steroid hormone biosynthesis. Moreover, we found nitrogen metabolism and primary bile acid biosynthesis in the late stage.

**Figure 12:**
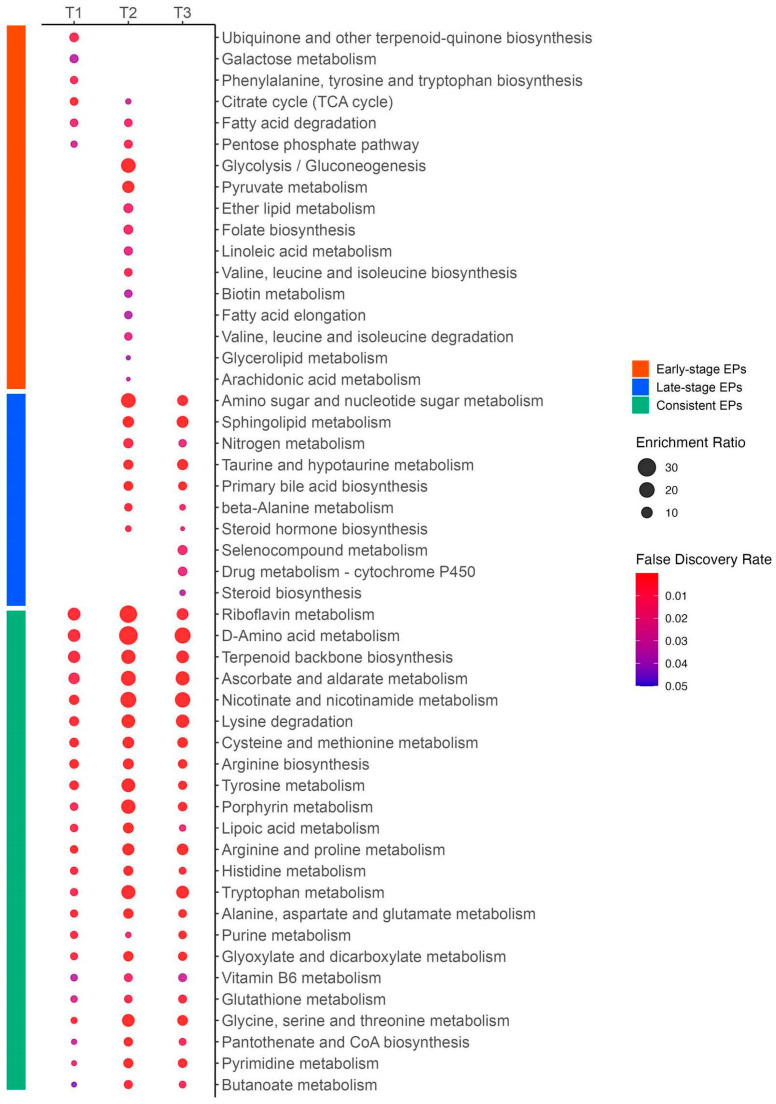
Enrichment ratios and false discovery rates of enriched pathways in early-stage, late-stage, and consistent categories. This figure was generated using MetaboAnalyst 6.0 (www.metaboanalyst.ca, accessed on 20 July 2024). Enrichment ratios were calculated as the number of observed metabolites divided by the number of expected metabolites within a specific metabolic pathway. The size of each circle represents the enrichment ratio, and the color indicates the false discovery rate.

Among the consistent EPs, we identified 11 pathways related to amino acid metabolism, including arginine and proline metabolism; arginine biosynthesis; cysteine and methionine metabolism; tyrosine metabolism; alanine, aspartate, and glutamate metabolism; glycine, serine, and threonine metabolism; lysine degradation; histidine metabolism; tryptophan metabolism; D-amino acid metabolism; and glutathione metabolism. We also found several vitamin-related pathways, such as nicotinate and nicotinamide metabolism (vitamin B3), riboflavin metabolism (vitamin B2), vitamin B6 metabolism, pantothenate and CoA biosynthesis (vitamin B5), and ascorbate and aldarate metabolism (vitamin C). Additionally, we discovered a few pathways related to nucleotide metabolism, including purine and pyrimidine metabolism. The EPs with these three categories are presented in Figure 12.

## 4. Discussion

In this study, we revealed the longitudinal differences in the dysregulated metabolism between survivors and non-survivors in patients with severe COVID-19. Specifically, we discovered metabolic dysregulations that improve, develop, or persist after the initiation of intensive care by identifying the early-stage, late-stage, and consistent DMs and EPs. Importantly, this hypothesis-generating study presents new hypotheses about which dysregulated metabolisms are possible biomarkers or therapeutic targets and when it is appropriate to test or treat them.

First, we discovered the association of the metabolic status with the ICU outcomes and time courses via PLS-ROG analysis, which was used to differentiate the time-dependent subclasses, and we identified 1-methyladenosine (m1A) as one of the top metabolites associated with ICU mortality. Notably, another methylated adenosine, *N*6-methyladenosine (m6A), which differs from m1A in its methylation position, was consistently higher in the non-survivors across all three time zones (data shown in Table S1). Both extracellular m1A and m6A can activate human adenosine 3 receptors (hA3R), acting as endogenous pro-inflammatory mediators. Of the two, m6A is known to be a more potent activator of hA3R, leading to the upregulation of genes involved in inflammatory cytokines and chemokines, such as interleukin-6 (IL-6), MCP-1, IL-1β, and CXCL2 [34]. Additionally, m6A mRNA methylation has been suggested as an epigenetic therapeutic target in conditions such as COVID-19, ARDS, and sepsis due to its role in regulating the gene expressions of signaling molecules in innate immunity, maintaining T-cell homeostasis, and controlling viral replication [35,36,37,38]. Elevated m6A methylation levels may, therefore, indicate the excessive translation of pro-inflammatory cytokines, prolonged T-cell activation, and increased viral replication. While the relationship between m6A methylation and increased extracellular m6A remains underexplored, the rise in both extracellular m1A and m6A could reflect greater immune dysregulation in non-survivors, suggesting their potential as biomarkers in severe COVID-19. Future studies are awaited.

Second, we identified the DMs and EPs in the early-stage, late-stage, and consistent groups. During the early phase, including T1 and/or T2, the glucose, amino acid, and FA metabolisms related to energy production differed. The enrichment pathway analysis detected the early-stage EPs of galactose metabolism, the citrate cycle, glycolysis/gluconeogenesis, pyruvate metabolism, and valine, leucine, and isoleucine degradation. We also detected the early-stage DMs of FA (24:5) and FA (17:3), with decreased blood levels in the non-survivors, and the early-stage EP of FA degradation. These findings could indicate that greater energy production is required for more excessive inflammatory responses and greater viral replication in the early phase in non-survivors compared with survivors [7,8,39]. Intriguingly, these differences existed only in T1 and/or T2 and became less pronounced in T3, which may suggest that while the energy production of survivors decreases as they recover their health in the late phase of T3, the energy production of non-survivors might inappropriately diminish due to muscle weakness and malnutrition development [40]. Further research is necessary to reveal the dysregulated energy production in severe COVID-19.

Third, during the late phase, including T2 and T3 or T3 alone, we detected several important differences in the sphingolipid, taurine, and tryptophan–kynurenine metabolism and steroid hormone synthesis. Sphingolipid has been proposed as a key molecule involved in endothelial injury. In our pathway analysis, we found that the sphingolipid metabolism was enriched during both T2 and T3. This increased dysregulation of sphingolipid metabolism in non-survivors is consistent with the previous findings, which report the development of endothelial injury in patients with severe COVID-19 around day 7 of hospitalization [41]. While the blood taurine levels did not significantly differ in T1 between the survivors and non-survivors, they were significantly more decreased in the non-survivors in T2 and T3. We also discovered that taurine was positively and strongly correlated with the platelet count. As taurine is known for its anti-inflammatory, antioxidant, and anti-platelet aggregation properties [42,43,44,45], greater taurine depletion may be associated with enhancements in inflammation, oxidative stress, and platelet aggregation in non-survivors. Studies are needed to determine whether taurine administration has therapeutic benefits for patients with severe COVID-19.

We identified the late-stage DMs of corticosterone, which had a significantly lower blood level in the non-survivors, and the late-stage EPs of steroid biosynthesis. Our findings showed the same trend as that in a previous study that presented lower corticosterone levels in severe patients than those in mild and moderate patients [46]. An autopsy study with COVID-19 patients discovered that the adrenal gland has signs of ischemic necrosis, cortical lipid degeneration, hemorrhaging, and focal adrenalitis [23], which might explain the decreased steroid synthesis in non-survivors, but further research is needed.

Tryptophan–kynurenine metabolism plays a crucial role in the immune system in COVID-19. We revealed that most of the metabolites in the tryptophan–kynurenine pathway showed increased blood levels in the non-survivors during T2 and T3, and that the tryptophan–kynurenine metabolism was consistently enriched with the increase in its enrichment ratio over time. Our results align with those of previous studies that revealed that the plasma kynurenine concentration is independently associated with hospital mortality in COVID-19 patients with ARDS [46], and that the kynurenine pathway is more activated in patients with severe COVID-19 than in those with mild and moderate COVID-19 [47,48,49]. Kynurenine exerts immunosuppressive effects mediated by aryl hydrocarbon receptor (AHR) signaling activation [50] and, through AHR activation, it suppresses antiviral immune responses driven by inhibiting NF-κB and IFN-I [14,51] in the innate immune system. While the anti-inflammatory response is necessary to resolve the acute-phase inflammation from infection, it might induce inappropriate immune suppression when it persists. Importantly, a previous study revealed that increased kynurenine blood levels in severe COVID-19 patients during the acute phase are associated with long-term outcomes, indicating the link between acute-phase metabolic abnormalities and long-term outcomes [24]. Our findings highlight the significance of the tryptophan–kynurenine pathway in COVID-19.

Fourthly, after excluding phenoxy acetic acid because it is derived from drugs, we identified three consistent DMs: homovanillic acid (HVA), N’-formyl kynurenine, and thromboxane B2 (TXB2). The HVA demonstrated higher blood levels across all three time zones in the non-survivors. HVA is an end product of dopamine metabolism that is produced by enzymes responsible for converting dopamine to homovanillic acid, such as monoamine oxidase (MAO) and catechol-O-methyltransferase (COMT). Few researchers have investigated HVA in COVID-19, but one previous study reported significantly higher HVA blood levels in deceased patients than those in survivors [47]. The consistently elevated HVA levels in our study may indicate progressively disrupted dopamine metabolism over time in non-survivors. In our pathway analysis, we identified a significant enrichment in tyrosine metabolism—a precursor to dopamine—across all three time zones. Additionally, we observed a positive correlation between HVA and inflammation markers such as C-reactive protein and the neutrophil-to-lymphocyte ratio. A previous study reported decreased dopamine blood levels and increased MAO expression in severe COVID-19 patients than those in healthy control and non-severe patients [15], and another preclinical study showed that SARS-CoV-2 may impair the dopamine production in dopaminergic neurons [48]. Some current studies propose disrupted dopamine metabolism as a potential mechanism underlying the neurological complications such as neuron senescence and immune-neuroendocrine interactions such as immunosuppression in COVID-19 [49,50]. These findings support the notion of disrupted dopamine metabolism in non-survivors of severe COVID-19. However, the mechanism of the increased HVA and dopamine metabolism disruption in COVID-19 remains underexplored, and further research is required.

Several previous studies have reported that the blood level of TXB2, an end product of arachidonic acid metabolism, is positively correlated with COVID-19 severity because of greater platelet activation and aggregation [19,51]. Notably, our findings showed consistently lower TXB2 blood levels in the non-survivors compared to the survivors, which might indicate the presence of additional pathologies in the arachidonic acid metabolism in non-survivors of severe COVID-19. Based on our findings, which showed decreased arachidonic acid blood levels in the non-survivors compared to the survivors (data are presented in Table S1), we hypothesize that this reduction leads to the depletion of the available arachidonic acid for thromboxane synthesis, resulting in lower TXB2 levels. To investigate this possible mechanism, further studies are needed.

Finally, metabolic alterations observed in post-COVID-19 syndrome (PCS), which is associated with various pathological conditions, such as lower back pain and insomnia, may contribute to our understanding of the metabolic dysregulation detected in this study [20,52,53]. Increased oxidative stress and disruptions in amino acid metabolism, including glutamine, arginine, and BCAAs, are linked to lower back pain in PCS. Oxidative stress exacerbates intervertebral disc degeneration, while amino acid metabolism disruptions impair muscle repair and the energy supply. In this study, we found the consistent enrichment of metabolic pathways such as glutathione metabolism, arginine biosynthesis, and aspartate and glutamate metabolism in the non-survivors across all three time zones, which may indicate increased intervertebral disc degeneration and impaired muscle repair, potentially associated with lower back pain in non-survivors of severe COVID-19. Additionally, the dysregulation of serotonin and dopamine metabolism has been associated with insomnia in PCS. In this study, serotonin was significantly decreased in T2 and T3, and HVA, the end product of dopamine metabolism, was consistently increased across all three time zones, which may suggest insomnia in non-survivors of severe COVID-19. However, the metabolic disruption associated with lower back pain and insomnia in severe COVID-19 is still under investigation, and further research is needed on this important issue.

## 5. Limitations

This study has several important limitations. First, the single-center design of this study may limit the generalizability of the results. However, our findings are consistent with several previous studies. A scoping review of patients with severe COVID-19 reported that the disruption in energy production through glycolysis and amino acid metabolism is associated with the disease severity [54]. Additionally, a previous systematic review reported that elevated levels of kynurenine and its metabolites are highlighted as a key feature of disease severity [55]. Future multicenter metabolomics studies on severe COVID-19 are needed to further validate and expand upon our findings. Second, our samples, which were only from severe COVID-19 patients with mechanical ventilation, may also limit the generalizability of our findings to patients with mild and moderate COVID-19. However, this approach enabled us to analyze a homogeneous cohort and obtain important insights into the severe COVID-19-specific pathophysiology. Third, the timing of the sample acquisition was not constant, and the sampling time zones were relatively wide due to the disruptions during the COVID-19 pandemic, preventing us from conducting more precise analyses with specific, more frequent sampling intervals. However, we have set clinically relevant time zones that align with the progression of severe COVID-19 to address this limitation. Fourth, we were unable to fully assess the dopamine metabolism disruption because dopamine was excluded from the analysis due to missing data in over 50% of the samples. Yet, as we addressed this point in the Section 4, our findings and several clinical and preclinical papers support the notion of the disrupted dopamine metabolism in severe COVID-19. More comprehensive metabolite analyses are necessary to achieve a more holistic understanding of the dopamine metabolism. Fifth, we excluded metabolites with missing values in more than 50% of the samples and imputed the missing values with one-fifth of the minimum detected metabolite value. This may have had a potential impact on the accuracy and reliability of the metabolite measurements, although the 50% threshold for missing data is widely used in metabolomics studies [56]. However, given the rarity and high value of our metabolomics data from patients with severe COVID-19, we prioritized retaining the maximum number of metabolites possible to gain comprehensive insights despite the potential trade-off in the accuracy. Sixth, while we focused on investigating the association of acute metabolic disruption (day 1 to day 30) with short-term outcomes, such as the ICU mortality, we cannot provide the relationship between the metabolic deterioration and long-term outcomes, such as the 60- and 90-day mortalities, due to our data availability limitation. We are currently collecting long-term outcome data for our patients and plan to address this critical aspect in future studies. Finally, our findings are preliminary and exploratory, and we are unable to make any assertions regarding a causal relationship between the identified metabolic pathways and ICU mortality or their implications for treatment recommendation. More robust analyses are required, such as prospective studies with larger sample sizes, validation in independent cohorts, and mechanistic experiments.

## 6. Conclusions

In this study, we investigated the longitudinal differences in metabolic dysregulation between survivors and non-survivors among patients with severe COVID-19 and revealed how the association between the metabolic status and ICU mortality changes over time. Our findings provide novel insights into the time-dependent variation in metabolic dysregulation in patients with severe COVID-19.

## Figures and Tables

**Table 1:** Comparison of baseline characteristics of mechanically ventilated patients with COVID-19 between survivors and non-survivors.

Characteristic	Overall n = 118	Survivor n = 93	Non-Survivor n = 25
Age (years)	61 (53, 73)	58 (52, 71)	71 (63, 78)
Female	22 (19%)	17 (18%)	5 (20%)
BMI (kg/m^2^)	24.6 (22.5,28.2)	24.7 (22.5,28.2)	24.0 (23.2,27.6)
SARS-CoV-2 Delta variant	25 (21%)	20 (22%)	5 (20%)
Onset to intubation interval (days)	9.1 (6.6, 11.6)	9.6 (7.6, 12.6)	7.6 (5.6, 10.6)
Comorbidity			
Hypertension	63 (53%)	47 (51%)	16 (64%)
Diabetes mellitus	39 (33%)	27 (29%)	12 (48%)
Ischemic heart disease	9 (8%)	3 (3%)	6 (24%)
Congestive heart failure	6 (5%)	2 (2%)	4 (16%)
Cerebrovascular disease	10 (8%)	8 (9%)	2 (8%)
Hemodialysis	4 (3%)	1 (1%)	3 (12%)
Dyslipidemia	27 (23%)	21 (23%)	6 (24%)
Asthma	7 (6%)	6 (6%)	1 (4%)
COPD	5 (4%)	4(4%)	1 (4%)
Interstitial pneumonia	2 (2%)	0 (0%)	2 (8%)
Lymphoma	1 (1%)	1 (1%)	0 (0%)
Cancer	6 (5%)	3 (3%)	3 (12%)
HIV	1 (1%)	1 (1%)	0 (0%)
Immunocompromised	8 (7%)	4 (4%)	4 (16%)
Severity			
APACHE II	15 (10, 20)	14 (10, 20)	19 (15, 23)
SOFA score	6 (4, 8)	5 (3, 7)	8 (6, 10)
Treatment			
NO therapy	56 (47%)	36 (39%)	20 (80%)
Prone positioning	71 (60%)	50 (54%)	21 (84%)
Steroid	99 (84%)	76 (82%)	23 (92%)
Favipiravir	43 (36%)	33 (35%)	10 (40%)
Remdesivir	84 (71%)	72 (77%)	12 (48%)
Tocilizumab	23 (19%)	20 (22%)	3 (12%)
Baricitinib	21 (18%)	18 (19%)	3 (12%)
mPSL pulse	32 (27%)	23 (25%)	9 (36%)
ECMO	14 (12%)	7 (8%)	7 (28%)
CRRT	23 (19%)	5 (5%)	18 (72%)
Complication			
Arrythmia	24 (20%)	14 (15%)	10 (40%)
DVT or PE	6 (5%)	3 (3%)	3 (12%)
Mediastinal emphysema	4 (3%)	1 (1%)	3 (12%)
Pneumothorax	6 (5%)	3 (3%)	3 (12%)
VAP	40 (34%)	25 (27%)	15 (60%)
BSI	33 (28%)	18 (19%)	15 (60%)
Clinical Outcome			
Intubation to ICU discharge interval (days)	12 (7, 21)	10 (7, 15)	27 (19, 40)
Discharge over 14 days	43 (36%)	24 (26%)	19 (76%)

Data presented as numbers (%) or medians (interquartile range). Abbreviations: COPD: chronic obstructive pulmonary disease; HIV: human immunodeficiency virus; APACHE II: acute physiologic assessment and chronic health evaluation II; SOFA: sequential organ failure assessment; mPSL: methylprednisolone; ECMO: extracorporeal membrane oxygenation; CRRT: continuous renal replacement therapy; DVT: deep venous thrombosis; PE: pulmonary embolism; VAP: ventilator-associated pneumonia; BSI: bloodstream infection.

## Data Availability

The data presented in this study are available upon request from the corresponding author due to confidentiality agreements and intellectual property restrictions with the collaborating company.

## Supplementary Materials

The following supporting information can be downloaded at https://www.mdpi.com/article/10.3390/metabo14120656/s1, Table S1: Results of volcano plot analysis comparing survivors and non-survivors at each time zone; Table S2: Results of linear mixed models for each metabolite; Table S3: Clinically laboratory test results between survivors and non-survivors at each time zone. Figure S1: Principal Components Analysis (PCA) did not clearly cluster the ICU outcome and the time-dependent subclasses; Figure S2: Partial Least Squares-Rank Order of Groups (PLS-ROG) showed the first and second PLS-ROG scores for the responsive variables; Figure S3: Partial Least Squares-Rank Order of Groups (PLS-ROG) identified metabolites that highly and significantly correlated with the PLS-ROG scores. A is a bar chart of Pearson’s correlation coefficients between the levels of each metabolite and the PLS-ROG1 score for the response variable. B is a bar chart of Pearson’s correlation coefficients between the levels of each metabolite and the PLS-ROG2 score for the response variable.
