# Supplementary material for: Longitudinal Metabolomics Reveals Metabolic Dysregulation Dynamics in Patients with Severe COVID-19
Source: Metabolites. 2024 Nov 25;14(12):656. doi: 10.3390/metabo14120656 (PMC11676849; doi:10.3390/metabo14120656)
Supplement: Supplementary file 1 [file metabolites-14-00656-s001.zip › Supplementay_figures_tables/Supplemental_Figures.pdf]

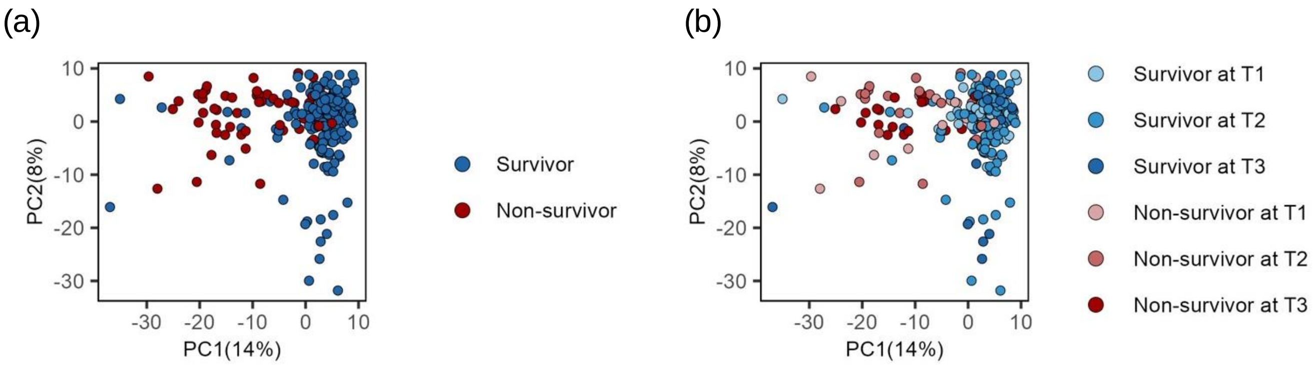

Supplemental Figure S1: Principal Components Analysis (PCA) did not clearly cluster the ICU outcome and the time-dependent subclasses. (a) PCA analysis illustrated the distribution of the first and second principal components with the color of the ICU outcome. (b) PCA analysis illustrated the distribution of the first and second principal components with the color of the time-dependent subclasses.

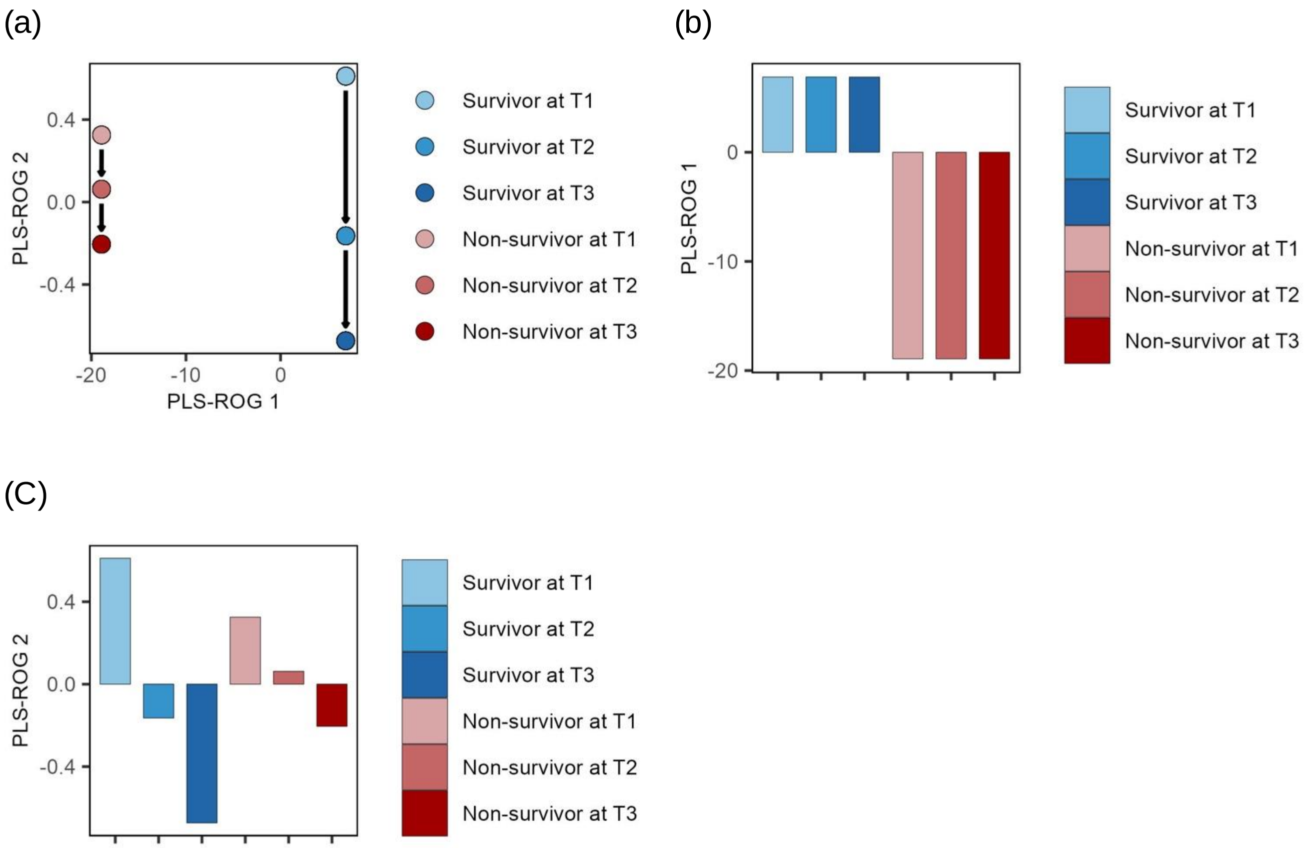

Supplemental Figure S2: Partial Least Squares-Rank Order of Groups (PLS-ROG) showed the first and second PLS-ROG scores for the responsive variables. (a) is a dot plot displaying the first and second PLS-ROG scores for the response variables colored by the time-dependent subclasses. (b) and (c) presented box plots of the first and second PLS-ROG scores for the response variables in the different time-dependent subclasses, respectively.

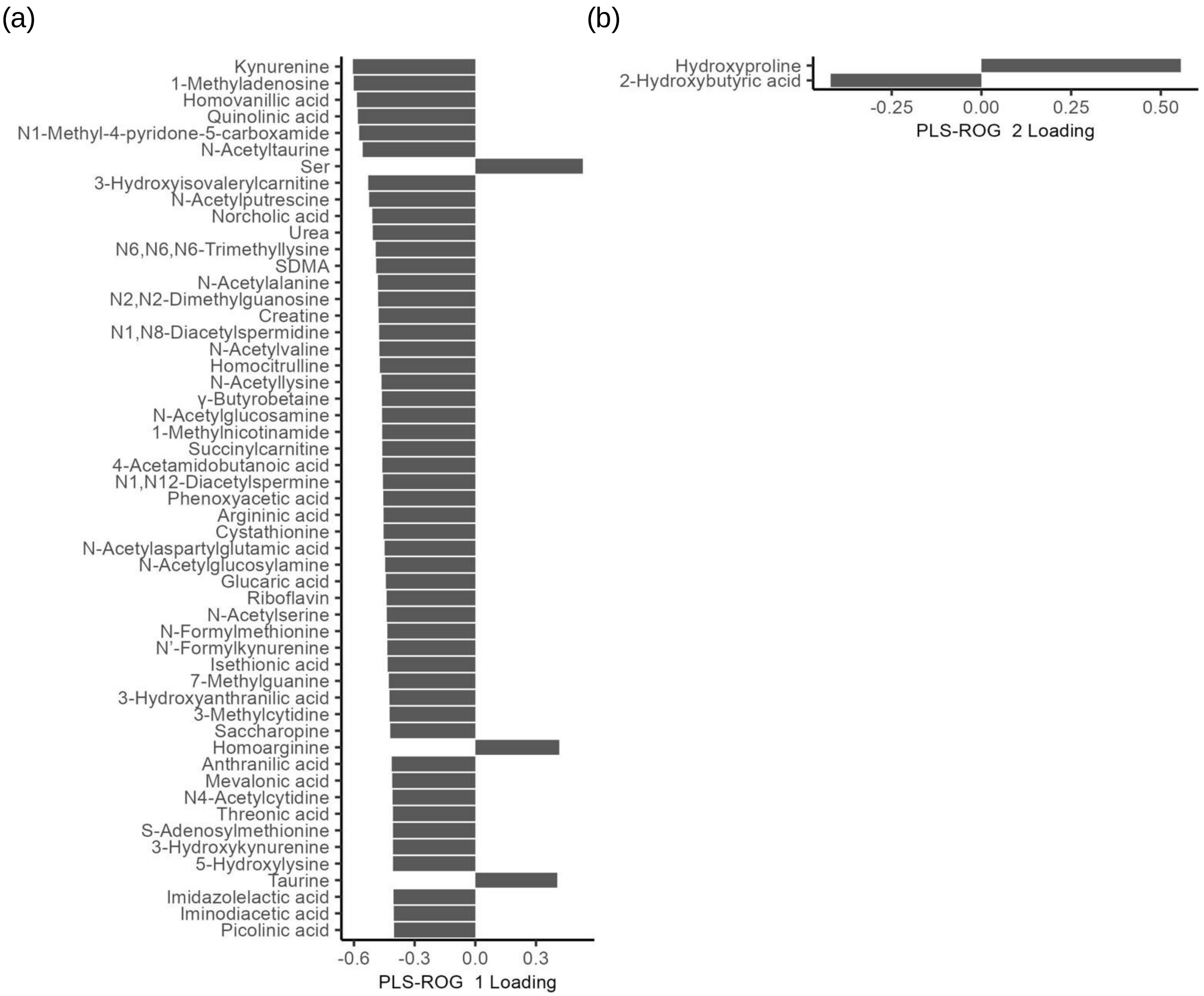

Supplemental Figure S3: Partial Least Squares-Rank Order of Groups (PLS-ROG) identified metabolites that highly and significantly correlated with the PLS-ROG scores. A is a bar chart of Pearson's correlation coefficients between the levels of each metabolite and the PLS-ROG1 score for the response variable. (a) showed 54 significant metabolites with absolute correlation coefficients over 0.4. B is a bar chart of Pearson's correlation coefficients between the levels of each metabolite and the PLS-ROG2 score for the response variable. (b) showed only two metabolites significantly correlated with the PLS-ROG2 score.
